# Unravelling the reasons for disproportion in the ratio of AOB and NOB in aerobic granular sludge

**DOI:** 10.1007/s00253-012-4126-9

**Published:** 2012-05-11

**Authors:** Mari K. H. Winkler, João P. Bassin, Robbert Kleerebezem, Dimitry Y. Sorokin, Mark C. M. van Loosdrecht

**Affiliations:** 1 Department of Biotechnology, Delft University of Technology, Julianalaan 65, 2628 BC Delft, The Netherlands; 2 COPPE-Chemical Engineering Program, Federal University of Rio de Janeiro, Rio de Janeiro, Brazil; 3 Winogradsky Institute of Microbiology, RAS, Moscow, Russia

**Keywords:** Aerobic granular sludge, Mixotrophic, Nitrite loop, AOB, NOB, PHA

## Abstract

In this study, we analysed the nitrifying microbial community (ammonium-oxidizing bacteria (AOB) and nitrite-oxidizing bacteria (NOB)) within three different aerobic granular sludge treatment systems as well as within one flocculent sludge system. Granular samples were taken from one pilot plant run on municipal wastewater as well as from two lab-scale reactors. Fluorescent in situ hybridization (FISH) and quantitative PCR (qPCR) showed that *Nitrobacter* was the dominant NOB in acetate-fed aerobic granules. In the conventional system, both *Nitrospira* and *Nitrobacter* were present in similar amounts. Remarkably, the NOB/AOB ratio in aerobic granular sludge was elevated but not in the conventional treatment plant suggesting that the growth of *Nitrobacter* within aerobic granular sludge, in particular, was partly uncoupled from the lithotrophic nitrite supply from AOB. This was supported by activity measurements which showed an approximately threefold higher nitrite oxidizing capacity than ammonium oxidizing capacity. Based on these findings, two hypotheses were considered: either *Nitrobacter* grew mixotrophically by acetate-dependent dissimilatory nitrate reduction (*ping-pong effect*) or a nitrite oxidation/nitrate reduction loop (*nitrite loop*) occurred in which denitrifiers reduced nitrate to nitrite supplying additional nitrite for the NOB apart from the AOB.

## Introduction

Nitrification is accomplished in a two-step process: oxidation of ammonium to nitrite by ammonium-oxidizing bacteria and/or archaea (AOB and AOA) and oxidation of nitrite to nitrate by nitrite-oxidizing bacteria (NOB). Both AOB and NOB are known to grow lithoautotrophically without the need for any organic compound. AOB belong to four genera in the Betaproteobacteria and Gammaproteobacteria and use oxygen as electron acceptor to oxidize ammonium in two steps by using AMO and HAO enzymes (Chain et al. 2003). NOB belong to five genera within different classes of the Proteobacteria and depend on AOB that generate their substrate (nitrite). NOB generate only two electrons by the oxidation of nitrite to nitrate, which is three times lower than the amount of electrons generated by AOB during oxidation of ammonium to nitrite. Due to ammonium activation by the AMO enzyme in the AOB metabolism, two electrons are not available for energy generation, and therefore the biomass yield of NOB is expected to be around two times lower than that of AOB per unit of nitrogen. This implies a theoretical NOB/AOB ratio of 0.5 (Aleem 1966, Ferguson 1982, Hagopian and Riley 1998, Hooper et al. 1997). The total numbers in NOB compared to AOB is expected to be even further lowered in systems where simultaneous nitrification/denitrification (SND) is taking place. Both nitrite and nitrate can be used as electron acceptor by denitrifying bacteria to generate nitrogen gas. If denitrification takes place mainly over nitrite, NOB would have to compete for nitrite with denitrifying organisms. In this case, it is likely that the NOB/AOB ratio will be even lower than 0.5 (Aleem 1966, Ferguson 1982, Hagopian and Riley 1998, Hooper et al. 1997). This trend will be true unless when the metabolism of NOB is changed in such a way that their biomass yield increases. This is possible if the growth of NOB does not only rely on the nitrite provided autotrophically by AOB, but also on other substrates (e.g. organic compounds), which suggests a mixotrophic metabolism of NOB. *Nitrobacter* is known to be capable of utilizing organic compounds and can grow by dissimilatory nitrate reduction (Freitag et al. 1987, Smith and Hoare 1968, Spieck and Bock 2005, Steinmüller and Bock 1976, Watson et al. 1989). Freitag et al. (1987) showed that a *Nitrobacter* biofilm culture performed oxidation of nitrite to nitrate on the surface of a silicon tubing from which oxygen diffused into the biofilm. The nitrate produced diffused away to the anoxic fraction of the biofilm where it was used by *Nitrobacter* as electron acceptor in the presence of an organic electron donor and converted back to nitrite. Due to this ‘ping-pong’ effect, the biomass yield increased three times compared to fully autotrophic cultures kept under aerobic conditions. Such an effect may also play an important role in biofilm systems in which multiple processes occur at the same time within different layers of the biofilm.

The microbial diversity of nitrifying bacteria has been extensively described for conventional activated sludge-based wastewater treatment systems. In these processes, the most common AOB found belong to the genus *Nitrosomonas* (Daims et al. 2009), while *Nitrospira* has been reported to be the most important NOB (Daims et al. 2001, Gieseke et al. 2003). Although the information about nitrifying bacteria in conventional systems is abundant, the information on the community of nitrifiers in aerobic granular sludge is sparse. To investigate the nitrifying community composition in granular biofilm, we decided to test the proportions between *Nitrospira*, *Nitrobacter* and *Nitrosomonas* by qPCR and FISH as well as to measure the microbial conversion rates (such as nitrite and ammonium oxidation rates) within different biofilm and flocculent sludge systems.

## Materials and methods

### Reactor set-up and operating conditions

A lab-scale aerobic granular sludge sequencing batch reactor (SBR) of 2.6 L was operated at 30 °C. Temperature was held constant by means of a thermostat, and the reactor was protected against cooling with a thermal isolation placed around the reactor. The operational cycle lasted 3 h and was divided into the following phases: 60-min anaerobic feeding from bottom of the reactor in a plug flow regimen; 112-min aeration period; 3-min settling and 5-min effluent withdrawal. Aeration and mixing were achieved through an air diffuser placed in the bottom of the reactors (airflow rate of 4 L/min). pH was kept at 7.0 ± 0.2 pH units by dosing 1 M NaOH or 1 M HCl. Dissolved oxygen (DO) concentration was controlled at 2.0 ± 3 mg O_2_/L by using two mass flow controllers (one for air and other for nitrogen gas). The volume exchange ratio and the hydraulic retention time (HRT) were 57 % and 5.2 h, respectively. Sludge retention time (SRT) was maintained around 30 days by periodically removing sludge from the reactor. The SRT calculation can be found elsewhere (Winkler et al. 2011a). The activation of influent/effluent and pH pumps was controlled by a bio-controller (Braun DCU4 coupled with MFCS control and data acquisition software). The synthetic feeding medium consisted of two solutions: (1) NaCH_3_COO·3H_2_O 63 mM, MgSO_4_·7H_2_O 3.6 mM, KCl 4.7 mM and (2) NH_4_Cl 35.4 mM, K_2_HPO_4_ 4.2 mM, KH_2_PO_4_ 2.1 mM and 10 mL/L trace element solution (Vishniac and Santer 1957). Per cycle, 150 mL was dosed from both solutions together with 1200 mL of tap water in order to achieve influent concentrations of 400 mg COD/L, 60 mg NH_4_-N/L and 20 mg P-PO_4_/L. Cycle tests under normal operational conditions were conducted, and samples were taken every 10–20 min during the aeration-mixing period to obtain the phosphate, ammonium, nitrite and nitrate profiles over an operational cycle.

### Anammox/nitration—CANON reactor

An autotrophic Anammox/nitration granular based on complete autotrophic nitrogen removal over nitrite (CANON) reactor was operated as described in previous research (Winkler et al. 2011b). The cycle consisted of a 60-min anaerobic feeding period followed by 112-min aeration, a 3-min settling period and 5-min effluent withdrawal. Mixing was performed with nitrogen gas during the first 60 min to keep anaerobic conditions. pH was kept at 7. The DO concentration was controlled at 1.0 mg O_2_/L in order to suppress the growth of NOB (Hao et al. 2005).

### Pilot-scale aerobic granular sludge and activated sludge systems

The pilot-scale aerobic granular sludge reactor and the activated sludge reactor, treating the same wastewater, were operated in parallel in a domestic wastewater treatment plant (Epe, The Netherlands). The pilot-scale granular sludge reactor (1.5 m^3^) was operated in a similar way as the lab-scale reactor. pH values were kept at around 7. The influent chemical oxygen demand (COD) and influent ammonium corresponded to approximately 600 mg/L and 50–100 mg N/L, respectively.

### Batch experiments under fully aerobic conditions

In order to compare the maximum ammonium and nitrite oxidation rates, batch tests were performed under fully aerated condition (DO kept at 100 % air saturation) with granular sludge from the lab-scale reactor. Representative biomass was taken at the end of the operational cycle (*t* = 180 min) and was pre-aerated for 2 h to assure that all ammonium was completely oxidized. Subsequently, the granules were washed with tap water whose temperature was adjusted to 30 °C (operating temperature of the reactor), sieved and gently crushed to prevent the occurrence of denitrification in the anoxic zones of the granules, which would interfere in the nitrite oxidation rate measurements. The crushed granules were distributed equally (based on the wet weight) to 250-mL flasks. Air was supplied to the batch flasks, which were filled with 50 mM Tris–HCl buffer (pH 7.0) and placed in a water bath kept at 30 °C. A pulse of either ammonium or nitrite solution was added in the beginning of the experiment in order to achieve initial concentrations of 50 mg N/L and 20 mg N/L, respectively. Samples were collected every 5–20 min and measured for ammonium, nitrite and nitrate. Biomass specific rates were obtained by linear regression of either ammonium or nitrite concentrations in time divided by the constant concentration of volatile suspended solids (g VSS/L) in the batch flask. The experiment was repeated several times over a period of 2 months.

### Batch experiments under fully anoxic conditions

Batch experiments were performed on biomass from the lab-scale reactor under anoxic conditions. Granules were collected from the aerobic granular sludge reactor after anaerobic feeding phase, i.e. after accumulation of polyhydroxybutyrate (PHB) by polyphosphate-accumulating organisms (PAOs) and glycogen-accumulating organisms (GAOs). Denitrifying PAOs and GAOs were actually the main active denitrifiers in the reactor, since no external organic carbon source was available during aeration period to be used as electron donor by other heterotrophic bacteria to accomplish denitrification in the anoxic zones of the granules. Granules were sieved and washed with tap water at 30 °C. Equal amounts of granules (based on the wet weight) were introduced in different 250-mL flasks filled with a Tris–HCl buffer (pH 7.0) containing the same minerals of the synthetic media fed to the reactor (except for acetate and phosphate). Nitrogen gas was supplied to each flask through porous diffusers in order to keep anaerobic conditions. A pulse of nitrate was given to obtain a final concentration of 30 mg N/L. From this point, samples were taken regularly every 10–20 min for determination of nitrate concentrations. Specific nitrate reduction rates could then be obtained by linear regression of nitrate concentration over time divided by the constant amount of VSS in the batch flask.

### Anoxic cycle tests

Anoxic cycle tests were also performed in the lab-scale reactor under fully anoxic conditions. In these tests, no air was supplied to the reactor, and anoxic conditions were achieved by supplying nitrogen gas instead of air through the porous diffuser. Nitrate was gradually added according to the denitrification rate, which was estimated based on the difference between the ammonium uptake rate and the nitrite/nitrate production rate obtained in the cycle tests performed under normal operation conditions (DO: 1.8 mg O_2_/L). Specific nitrate reduction rates were obtained by linear regression of the nitrate uptake in time divided by the amount of VSS in the reactor.

### Analytical measurements

Ammonium-nitrogen (NH_4_-N), nitrate-nitrogen (NO_3_-N) and nitrite nitrogen (NO_2_-N) were measured by means of a flow injection analyzer (QuikChem 8500, Lachat Instruments, Inc.). Phosphate (PO_4_-P) was quantified by Hach Lange cuvette tests (LCK 350). Biomass concentration, in terms of volatile suspended solids (VSS), was determined as described in previous research (Winkler et al. 2011a).

### Sample collection for qPCR

Samples were taken over a period of 1 year from four different reactors: two lab-scale aerobic granular sludge reactors (one operated as a conventional granular system and other as an autotrophic partial-nitritation/Anammox (CANON) reactor (Winkler et al. 2011b), a pilot-scale aerobic granular sludge sequencing-batch reactor and an activated sludge reactor. All samples were checked with qPCR for their AOB/NOB ratios.

### Quantitative PCR (qPCR)

Primers and PCR conditions are listed in Table 1. Primers were checked in the ARB database as well as in the Ribosomal Database Project (RDP). All samples were measured in triplicates. DNA extraction was conducted with the UltraClean™ Microbial DNA Isolation Kit (MO BIO Laboratories, USA). First, a regular PCR was performed followed by a purification step with the QIAquick PCR Purification Kit (250) (Germany, QIAGEN). The PCR product was used for a qPCR procedure with a variable primer concentration and 25 μM iQ SYBR® Green Supermix (BIO-RAD Laboratories, USA) (Table 1). iCycler iQ™ Multi-Color Real-Time PCR Detection System (BIO-RAD Laboratories, USA) was used to run the qPCR assays. All primers were optimized with a gradient qPCR. The resulting conditions, primer concentration as well as the DNA used as a standard for the qPCR are listed in Table 1. A picogreen protocol was used to determine the amount of DNA template in order to normalize all *C*_T_ values to 5 ng DNA by using a Pico Green dsDNA Quantitation Kit (Molecular Probes Inc., USA). Δ*C*_T_ was calculated by the following equation: Δ*C*_T_ = *C*_T(ref)_ – *C*_T(target)_ (Zhang et al. 2009). For determination of the ratios of a specific organism (e.g. target = AOB) to the total community (ref), the following equation was applied: ${\text{rati}}{{\text{o}}_{{{\text{target/ref}}}}} = {{2}^{{\Delta {{C}_{\text{T}}}}}}$. For construction of a ratio between NOB and AOB, the calculated ratio of AOB was divided by the calculated ratio of NOB.

**Table 1:** Primers, qPCR conditions, primer concentrations and references

Primer	Primer con. [μM]	qPCR conditions	Standard	Reference
CTO189F A/B & CTO189F C CTO654R	0.2	94°/2′ [94°/0.30′ 61°/1′ 72°/0.45′] × 30 52°/10′ 10°/∞	*Nitrosomonas europaea*	(Kowalchuk et al. 1997)
NTSPA1026F NTSPA1026R	0.2	94°/1′ [94°/0.5′ 45°/0.2′ 72°/0.3′80°/0.25′] × 40 72°/10′ 10°/∞	*Nitrospira defluvii*	(Juretschko et al. 1998)
NTS232F/NTS 1200R	0.2	95/5 m, (95/40 s, 55–48/30 s, 72/60 s)*35, 72/10 min, 12/∞	*Nitrospira defluvii*	(Degrange et al. 1997, Lim et al. 2008)
FGPS872/FGPS 1269	0.2	95/3 m, (95/60 s, 50/60 s, 72/60 s)*35, 72/3 min, 12/∞	*Nitrobacter*	
AMX1066RAMX818F	0.3	95°/10′ [94°/0.15′ 61°/0.1′ 68°/0.25′ 80°/0.25′] × 40 52°/10′ 10°/∞	Sludge from wwtp^a^	(Tsushima et al. 2007)
Bac341F	0.355	95°/6′ [95°/0.30′ 55°/0.4′ 52°/0.4′80°/0.25′] × 40	Sludge from wwtp^b^	(Muyzer et al. 1993)
Bac905r		52°/5′10°/∞	Sludge from wwtp^b^	(Weisburg et al. 1991)

Primers used and their concentrations as well as the standard used. qPCR conditions show temperature and cycle length for denaturing, annealing, elongation and cooling steps

^a^Anammox enriched sludge from Anammox reactor in Rotterdam, The Netherlands

^b^Sludge from the aerobic granular sludge pilot plant in Epe, The Netherlands

### FISH

Granules from the lab- and pilot-scale aerobic granular sludge reactors were fixed in 4 % paraformaldehyde. Granules where embedded in a tissue freezing medium (Tissue Freezing medium, Leica Microsystems), hardened by freezing (−20 °C) and cut in the frozen state with a microtome-cryostat (Leica CM1900-Cryostat) into 25-μm thin slices. Dried slices were kept on a microscopic glass slide, and FISH was performed for determination of PAOs, GAOs, AOB and NOB (Fluos) populations in the same manner as reported by Winkler et al. (2011b). Sequences are listed in Table 2. Samples were analysed with an epifluorescence microscope (Axioplan 2, Zeiss) equipped with filters suited for Cy3, Cy5 as well as Fluos.

**Table 2:** Oligonucleotide probes, primers target microorganisms and references used in this study

Probes	Sequence (from ′5 to ′3)	Specificity	Reference
NOB mix			
Ntspa662	GGAATTCCGCGCTCCTCT	*Nitrospira-*like organisms	(Daims et al. 2001)
NIT1035	CCTGTGCTCCATGCTCCG	*Nitrobacter*	(Wagner et al. 1996)
AOB mix			
NSO190	CGATCCCCTGCTTTTCTCC	All AOB	(Mobarry et al. 1996)
NSO1225	CGCCATTGTATTACGTGTGA	All AOB	(Mobarry et al. 1996)
PAO mix			
PAO 462	CCGTCATCTACWCAGGGTATTAAC	Most *Accumulibacter*	(Crocetti et al. 2000)
PAO 651	CCC TCTGCCAAACTCCAG	Most *Accumulibacter*	(Crocetti et al. 2000)
PAO 846	GTTAGCTACGGACTAAAAGG	Most *Accumulibacter*	(Crocetti et al. 2000)

Probes from NOBs were tagged with the fluorescent dye Fluos (green) Anammox with Cy3 (red) and AOBs with Cy5 (blue). For analysis probes of one target group were mixed

### Biomass yields

The estimated autotrophic community composition was based on the biomass yields on ammonium (AOB) or nitrite (NOB) (*Y*_X/N_). For mixotrophic growth of NOB on acetate, a biomass yield from heterotrophs was assumed (*Y*_X/HAC_) (Table 3). The theoretical amount of COD needed to increase the NOB/AOB ratio (ratio including autotrophic NOB) to 3 was calculated to be 37 mg COD/L. For the conversion from COD to VSS, a factor of 1.4 was used (Scherer et al. 1983).

**Table 3:** Biomass yields for AOB and NOB, and an assumed heterotrophic biomass yield for mixotrophic NOB and the corresponding biomass concentration and relative community composition according to consumed substrate (37 mg COD/L; 60 mg NH_4_-N/L)

	*Y*x/Hac	*Y*x/N	mg VSS/L	Community composition [%]		Reference for yields
AOB	–	0.13	8	67^a^	25^b^	(Blackburne et al. 2007b)
NOB	–	0.07	4	33^a^	12^b^	(Blackburne et al. 2007a)
Mixotropic NOB	0.4	–	21	–	63^b^	(Beun et al. 2001)
NOB/AOB ratio				0.5	3	

Calculations based on assumption of a) autotrophic growth of nitrifiers and b) mixotrophic growth of NOB on 37 mg COD/L. ${{Y}_{{{\text{x/ Hac}}}}}\left[ {C{\text{mol}}/C{\text{mol}}} \right]$_;_ ${{Y}_{{{\text{x/N}}}}}\left[ {C{\text{mol}}/N{\text{mol}}} \right]$

## Results

### Reactor cycle measurement

**Fig. 1:**
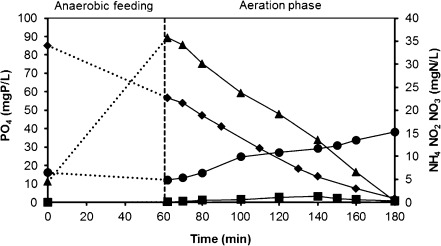
Typical cycle test showing the profiles of (♦) ammonium, (●) nitrate and (■) nitrite (right *y*-axis) and (▲) phosphate (left *y*-axis). The starting ammonium and phosphate concentrations depicted at time 0 were calculated based on the influent concentration (60 mg NH_4_-N/L and 20 mg PO_4_-P/L) and the dilution in the reactor. Nitrite and nitrate concentrations at time 0 were calculated based on their concentrations in the end of the cycle and the dilution in the reactor. The *dashed lines* displayed from the beginning until the end of the feeding period do not represent data points. They were inserted to better visualize the amount of phosphate release and ammonium adsorption observed during the anaerobic phase

Figure 1 shows a typical cycle test performed during the operation of the lab-scale reactor, when a steady-state condition was reached. The first sample was collected 2 min after the aeration phase has started (*t* = 62 min) to allow sufficient mixing. During the anaerobic feeding period (data not shown), all acetate is converted to intracellular polymers (e.g. polyhydroxybutyrate—PHB) by PAOs or GAOs leaving no dissolved organic carbon for the subsequent aerobic period. During anaerobic feeding, phosphate is released by PAOs and is subsequently taken up by the same microorganisms during the aerobic period. After the feeding period, PAOs and GAOs degrade internally stored PHB and use the generated energy for growth (Smolders et al. 1994). Ammonium concentration after anaerobic feeding was observed to be lower than expected based on the influent concentration and the dilution in the reactor. This fact is due to ammonium adsorption, phenomena that was investigated in a separate study (Bassin et al. 2011). Ammonium removal was practically constant and amounted to around 100 % during the whole experimental period. Nitrite concentrations were very low along the operational cycle (lower than 0.3 mg N/L), although an accumulation of nitrate was observed in the end of the cycle. The nitrate remaining after effluent discharge was present in the subsequent cycle although diluted with the following feed.

### Batch experiments under fully aerobic conditions

**Fig. 2:**
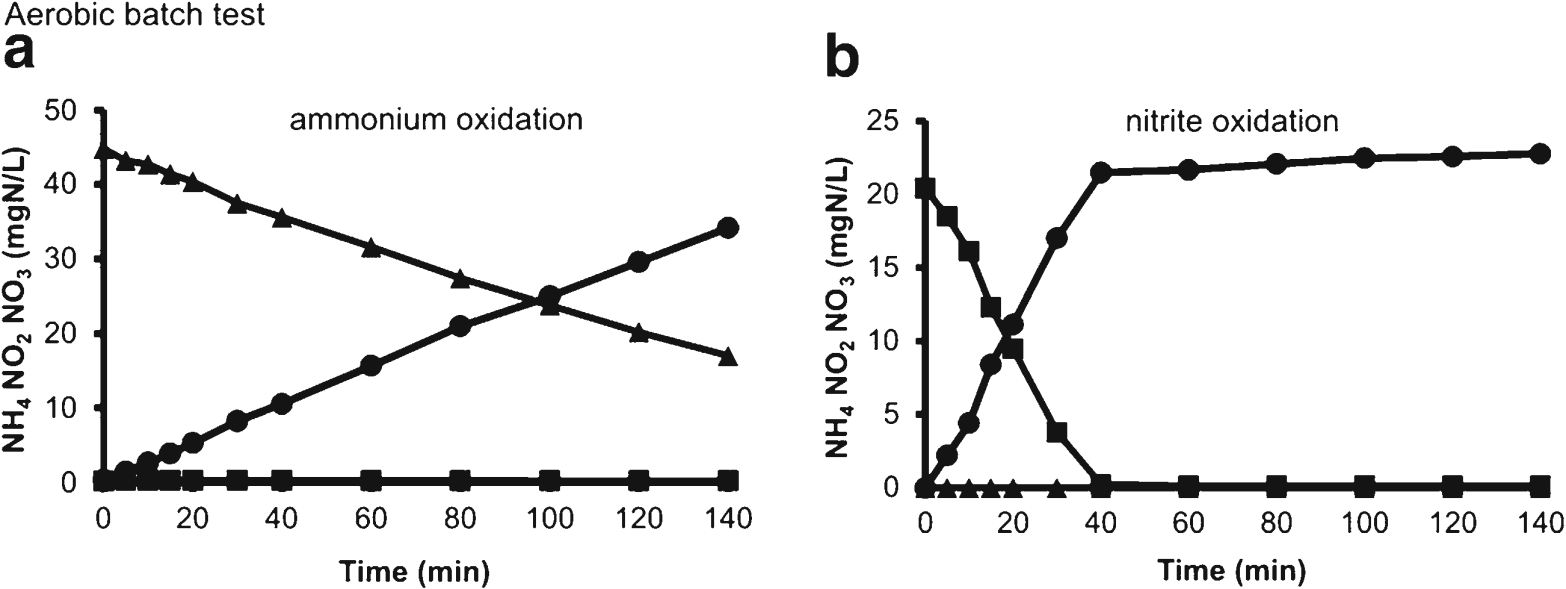
Aerobic batch tests dosing either ammonium (**a**) or nitrite (**b**). Legend description: ammonium (▲), nitrite (■) and nitrate (●)

Batch experiments with crushed granules (to eliminate oxygen diffusion limitation) under fully aerobic conditions (DO kept at 100 % air saturation) were performed to compare the maximum ammonium- and nitrite-oxidizing activities. The ammonium, nitrite and nitrate profiles of the aerobic tests with ammonium and nitrite dosage are illustrated in Fig. 2a, b, respectively. Results from this set of experiments using the same biomass revealed an approximately threefold higher nitrite uptake rate (6.0 mgNO_2_-N/gVSS/h) than the ammonium uptake rate (2.3 mg NH_4_-N/gVSS/h). Since denitrification was prevented by using crushed granules (therefore eliminating the anoxic zones within the granules), all nitrite consumption was due to nitrite-oxidizing activity. Repeated experiments showed a good reproducibility. The results shown in Fig. 2 are in line with FISH and q-PCR observations, which showed a higher NOB/AOB ratio in aerobic granular biomass compared to the calculated theoretical ratio of 0.5, assuming autotrophic nitrifying growth (Table 3).

### Batch experiments and cycle tests under fully anoxic conditions

**Fig. 3:**
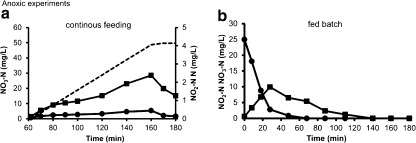
Anoxic experiments dosing nitrate (**a**) in a continuous feeding regime and (**b**) pulse feeding regime. Legend description: nitrite (■), nitrate (●). The *dashed line* shows the calculated amount of nitrate added

During normal reactor operation, nitrification and denitrification processes occur simultaneously. Therefore, it is difficult to distinguish the amount of nitrite oxidized by NOB and the amount of nitrite reduced by denitrifiers. In order to have a better insight into the denitrification potential in the granular sludge reactor, two types of experiments were carried out under anoxic conditions: anoxic batch test in the aerobic granular sludge reactor with a continuous supply of nitrate instead of oxygen during the aeration (mixing) phase (Fig. 3a) and a batch experiment in which nitrate was added as a pulse in the beginning of the test (Fig. 3b). The anoxic cycle test has shown that no nitrite accumulated over the whole experiment, a result similar to that observed under normal reactor operating conditions (see cycle test in Fig. 1). In the batch experiment in which nitrate was supplied as a pulse (high concentration in the beginning of the test), nitrate reduction was accompanied by nitrite accumulation. After nitrate depletion, the accumulated nitrite was reduced.

### Relative quantification of AOB and NOB by qPCR

**Fig. 4:**
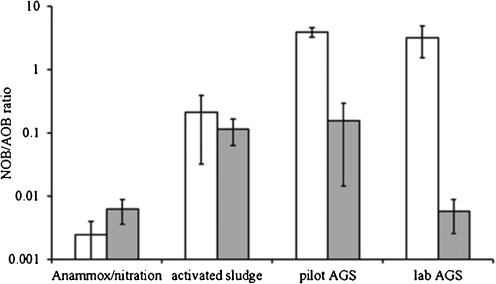
Average of NOB/AOB ratios built on the basis of 16S rRNA gene analysis of *Nitrobacter*/AOB (□) and *Nitrospira*/AOB  of (left to right) autotrophic nitritation Anammox (CANON) reactor, activated sludge, aerobic granular sludge pilot plant and aerobic granular sludge lab reactor. *Error bars* indicate maximum and minimum observed ratios

To study the nitrifying population within the aerobic granular sludge, a comparative study between the amount of AOB and NOB was performed by means of qPCR. Ratios between NOB and AOB were constructed for samples collected from an autotrophic CANON/Anammox reactor, a conventional treatment plant, an aerobic granular sludge pilot plant and a lab-scale aerobic granular sludge reactor (Fig. 4). Both assays from aerobic granular sludge samples showed high NOB/AOB ratios (3–4), a moderate ratio in the conventional treatment plant (0.2 ± 0.1) and low ratios in the CANON reactor (0.004 ± 0.002). *Nitrobacter* was the main NOB present in aerobic granular sludge. In the conventional treatment plant, both *Nitrospira* and *Nitrobacter* coexisted in roughly equal amounts.

### FISH

**Fig. 5:**
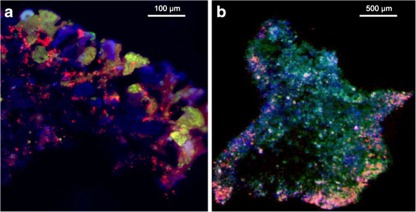
Microscopic FISH image on sliced granule showing **a** AOB (*green*), NOB (*red*) and PAO (*blue*) as well as **b** nitrifiers (mix of AOB and NOB) (*red*), PAO (*blue*) and GAO (*green*)

In wastewater treatment systems, nitrifiers are expected to account for 1–3 % of the total community composition due to their low growth yield. Therefore, FISH was also conducted with PAOs and GAOs to have a reference for the heterotrophic community (Fig. 5b). The FISH pictures showed the same trend as the qPCR results, i.e. an elevated number of NOB. Moreover, it also showed that NOB grew deeper in the biofilm when compared to AOB (Fig. 5a).

### Biomass yields

In order to calculate how much COD is needed to increase the NOB/AOB ratio to 3, we assumed that NOB grew mixotrophically on organic acids with nitrate as electron acceptor and we considered a biomass yield similar to general heterotrophs. Calculations showed that a theoretical amount of 37 mg COD/L is needed to elevate the expected autotrophic NOB/AOB ratio of 0.5 by a factor 6 (Table 3). This amount of COD is less than 10 % of the total acetate fed into the system (400 mg COD/L).

## Discussion

### Introduction

Our research demonstrated an elevated NOB/AOB ratio (higher than the expected ratio of 0.5 based on autotrophic ammonium and nitrite oxidation) in heterotrophic aerobic granular sludge from a pilot plant and a lab-scale reactor using FISH, qPCR analysis and activity batch tests. In contrast, qPCR measurements on samples from the conventional treatment plant operated in parallel with the aerobic granular sludge pilot reactor did not show elevated NOB levels. Moreover, the negative control samples collected from a CANON reactor revealed expectedly low NOB/AOB ratios (i.e. ca. 100-fold lower than AOB). A qPCR on ammonium-oxidizing archaea was conducted, but no signal could be detected (data not shown). *Nitrobacter* and not *Nitrospira* was the dominant NOB in aerobic granular sludge pilot plant and lab-scale reactor. This is interesting since *Nitrospira* has been identified as the dominant NOB in activated sludge systems (Daims et al. 2001; Gieseke et al. 2003).

**Fig. 6:**
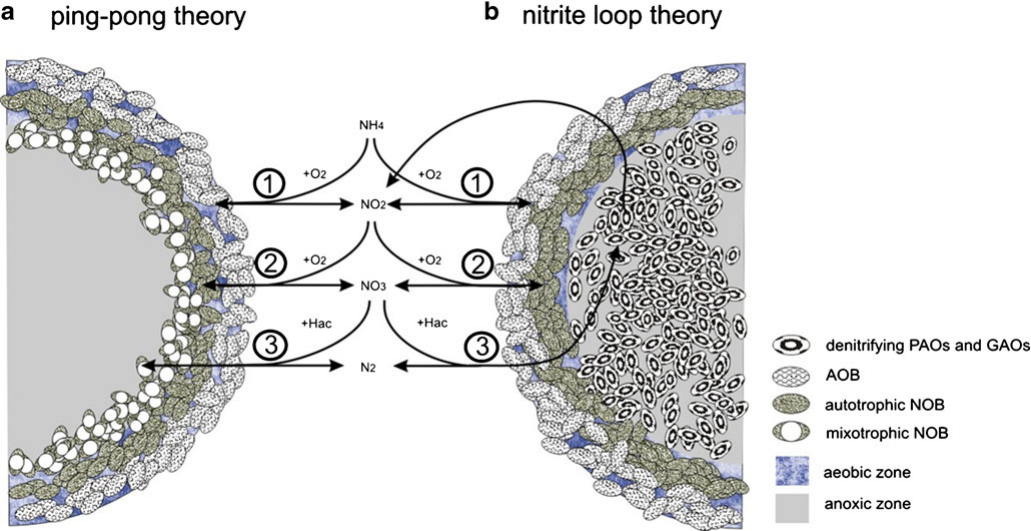
Schematic view of **a** the ping-pong theory and **b** nitrite loop theory. Both theories have step 1) oxidation of ammonium to nitrite via ammonium oxidizing bacteria (AOB) and step 2) oxidation of nitrite by autotrophic nitrite oxidizing bacteria (NOB) in common. In case of the ping-pong effect (**a**) the third step assumes that organotropic NOB take up acetate as PHB and hence outcompete general heterotrophs for organic acids. *Nitrobacter* can then grow on acetate hence increasing its cell yield. In case of the nitrite loop (**b**) it is assumed that denitrifying PAOs and GAOs incompletely reduce nitrate with acetate only until nitrite by which it can be reoxidized by NOBs, which in turn can grow uncoupled from the nitrite supply of AOBs

Based on these experimental results, we propose two possible mechanisms to explain our unexpected observations regarding the NOB/AOB ratio. The first assumption refers to the ping-pong effect (Fig. 6a). In this process, excess nitrate is reduced by mixotrophic *Nitrobacter* using acetate as electron donor and carbon source, and therefore the growth of *Nitrobacter* is uncoupled from the direct nitrite supply of the AOB. The second theory is referred to as the ‘nitrite loop’, in which a nitrite oxidation/nitrate reduction loop takes place in the granules (Fig. 6b). If denitrification would be incomplete (i.e. from nitrate to nitrite only), accumulated nitrite could be reused by NOB, which would partly uncouple their growth from AOB. Both theories can be relevant especially in biofilm systems, in which simultaneous oxidation of nitrite to nitrate (by NOB) and reduction of nitrate to nitrite (by denitrifiers or mixotrophic NOB) can occur.

### Ping-pong theory

*Nitrospira* is suggested to outcompete *Nitrobacter* at low nitrite concentrations due to the lower nitrite half-saturation constant of the former microorganisms (Blackburne et al. 2007a). Very low concentrations of nitrite were detected during normal operation of the lab- and pilot-scale aerobic granular sludge reactors indicating that conditions are more likely to be in favour of *Nitrospira*. However, the aerobic granular sludge system was dominated by *Nitrobacter* (Figs. 4 and 5). Numerous researches have shown experimentally as well as by mathematical modelling that oxygen penetration is restricted to the outer rim (< 100 μm) of the granule (Nielsen et al. 2005; Picioreanu et al. 2004). Conversely, according to our FISH pictures, *Nitrobacter* grew up to 300 μm deep. AOB were only present in the outer layer until approximately 100 μm. This suggests that NOB might have used another electron acceptor than oxygen for growth. In our experimental setup, all acetate supplied was stored as PHB by PAOs and GAOs during anaerobic feeding. However, during the feeding period with acetate, remaining nitrate from the previous cycle is available in the granules which may allow for heterotrophic denitrification by the NOB. It can also not be excluded that *Nitrobacter* present in the anoxic regions grows heterotrophically on soluble microbial products or decay products from the active biomass. Based on our calculations assuming that mixotrophic NOB would grow on organic acids with a biomass yield typical for heterotrophs, we found that only 37 mg COD/L is needed to increase the NOB/AOB ratio to the observed one of approximately 3 (Table 3). In a previous research in which a single *Nitrobacter* culture was studied, aerobic oxidation of nitrite to nitrate was shown to occur on the surface of a silicon loop while produced nitrate diffused away to the anoxic parts of the biofilm (Freitag et al. 1987). In the anoxic environment, nitrate was used as electron acceptor with a provided organic electron donor and was converted back to nitrite. During this ping-pong effect, *Nitrobacter* carried out dissimilatory nitrate reduction and was reported to store PHB (Freitag et al. 1987).

The accumulation of reserve polymers (e.g. PHB and poly-P) has been experimentally proven in pure *Nitrobacter* culture experiments (Bock 1976; Freitag et al. 1987; Pope et al. 1969; Van Gool et al. 1971; Watson et al. 1989). Additionally, *Nitrobacter* has the genes *NarJ* and *NarI* confirming their capability of dissimilatory nitrate reduction (Starkenburg et al. 2006, 2008). From an ecological perspective, it is advantageous for bacteria to rapidly store organic carbon as energy rich PHA and use it for growth when no external substrate is available (i.e. under starvation conditions). This particular metabolism is often observed in sequencing batch processes, in which microorganisms are subjected to alternating phases of high and low substrate availability, designated as feast and famine regimes, respectively (Jiang et al. 2011; Johnson et al. 2010). The activated sludge process is mainly based on continuously fed systems, whereas aerobic granular sludge is primarily operated in SBRs, which could indeed stimulate the organisms (e.g. *Nitrobacter*) to store intracellular polymers to balance their growth (Van Loosdrecht et al. 1997). In our aerobic granular sludge system, the fast uptake of acetate could give *Nitrobacter* an extra competitive advantage. *Nitrosomonas europea* (AOB) has also all enzymes of the tricarboxylic acid (TCA) cycle but does not synthesize PHB as storage products, and there is also no experimental evidence that *Nitrosomonas* can generate PHB from organic acids (Abeliovich and Vonshak 1992; Chain et al. 2003). *Nitrobacter* could hence increase its proportion in a microbial community relative to *Nitrosomonas* and even general heterotrophs by using organic acids and storing reserve polymers. We suspect that if *Nitrobacter* used its mixotrophic capability, it is more probably due to its ability to grow by heterotrophic nitrate reduction in anoxic environment rather than by an aerobic metabolism.

### Nitrite loop theory

To support the nitrite loop theory, nitrite needs to become available in the biofilm or even in the bulk, which in turn can be reused by NOB. Such an accumulation was not measurable during normal reactor operation (Fig. 1). However, in a batch test in which we dosed twice the amount of nitrate denitrified under normal operation (Fig. 3a), an accumulation of nitrite was observed. In the denitrification pathway, seven enzymes catalyse the facultative respiratory pathway, in which nitrate (NO_3_), nitrite (NO_2_), nitric oxide (NO) and nitrous oxide (N_2_O) are reduced to nitrogen gas (N_2_) (Philippot 2002; Zumft 1997). Earlier studies have shown that the reduction of soluble NO_2_^−^ to gaseous NO by nitrite reductases (*NirK* or *NirS*) can be the rate limiting step in denitrification in certain types of bacteria (Firestone et al. 1979). The measured nitrite accumulation designates that additional nitrite might become available within the biofilm during reactor operation hence enabling the nitrite loop. In a biofilm system, all oxidation and reduction processes occur within multi-structural layers harbouring all microorganisms. Since nitrification and denitrification processes occur simultaneously, it is troublesome to measure them separately. In conventional wastewaters treatment systems, the nitrite loop is unlikely to occur, since nitrification and denitrification processes are usually carried out in different compartments. In pre-denitrification systems, the anoxic compartment is rich in organic carbon whereas the aerobic compartment has only little organic carbon. This is supported by the qPCR results showing lower NOB/AOB ratios for conventional treatment plant as opposed to higher NOB/AOB ratios observed in both pilot- and lab-scale aerobic granular sludge reactors (Fig. 4).

In this study, several methodologies (FISH, qPCR and activity tests) were used to investigate and understand the unexpected elevated NOB/AOB ratio in aerobic granular sludge systems. We observed that the NOB population in the granular systems was dominated by *Nitrobacter*.

Two hypotheses were proposed: either *Nitrobacter* have grown mixotrophically by acetate-dependent dissimilatory nitrate reduction (*ping-pong effect*) or a nitrite oxidation/nitrate reduction loop (*nitrite loop*) have happened in which denitrifying bacteria reduced nitrate to nitrite providing additional nitrite for the NOB apart from the AOB. The disproportion of the amount of AOB and NOB in granular sludge should be investigated further to confirm the hypothesis made in this work.
